# Tales from the crypt: a parasitoid manipulates the behaviour of its parasite host

**DOI:** 10.1098/rspb.2016.2365

**Published:** 2017-01-25

**Authors:** Kelly L. Weinersmith, Sean M. Liu, Andrew A. Forbes, Scott P. Egan

**Affiliations:** 1 Department of BioSciences, Rice University, MS-140, 6100 Main Street, Houston, TX 77005, USA; 2 Department of Biology, University of Iowa, Iowa City, IA 52242, USA

**Keywords:** manipulation, *Bassettia pallida*, crypt-keeper wasp, *Euderus set*, gall wasps, parasitoids

## Abstract

There are many examples of apparent manipulation of host phenotype by parasites, yet few examples of hypermanipulation—where a phenotype-manipulating parasite is itself manipulated by a parasite. Moreover, few studies confirm manipulation is occurring by quantifying whether the host's changed phenotype increases parasite fitness. Here we describe a novel case of hypermanipulation, in which the crypt gall wasp *Bassettia pallida* (a phenotypic manipulator of its tree host) is manipulated by the parasitoid crypt-keeper wasp *Euderus set*, and show that the host's changed behaviour increases parasitoid fitness. *Bassettia pallida* parasitizes sand live oaks and induces the formation of a ‘crypt’ within developing stems. When parasitized by *E. set*, *B. pallida* adults excavate an emergence hole in the crypt wall, plug the hole with their head and die. We show experimentally that this phenomenon benefits *E. set*, as *E. set* that need to excavate an emergence hole themselves are about three times more likely to die trapped in the crypt. In addition, we discuss museum and field data to explore the distribution of the crypt-keeping phenomena.

## 1. Introduction

Many animals are infected by parasites that modify host phenotype in ways that benefit the parasites while harming the host [1–4]. This phenomenon is known as parasite manipulation, and when manipulation is occurring the host phenotype is an extended phenotype of the parasite [5]. Examples include parasites that change host behaviour or appearance in ways that increase the host's risk of being eaten by the next host in the parasite's life cycle [6–8], and parasitoids that induce their insect hosts to commit ‘suicide’ by jumping into water so the parasitoid can find mates and complete the aquatic phase of its life cycle [9,10]. These parasites can have important ecological implications [11,12], and by understanding how parasites induce complex changes in host phenotype, we are exploring novel links between the immune system, nervous system and behaviour [13].

The observation that the manipulation appears to be fairly widespread may lead one to wonder—are also the manipulators manipulated? That is, how common is hypermanipulation? One example of a manipulated manipulator is the fungus *Ophiocordyceps unilateralis*, which manipulates its arboreal ant host (*Camponotus leonardi*) into leaving its nest in search of a location that is amenable to fungal reproduction [14]. *Ophiocordyceps unilateralis* is itself susceptible to castration by another fungus [15]. Additionally, the dipteran tree parasite *Masakimyia pustulae* manipulates its tree host into producing leaf galls, and *M. pustulae* may be manipulated by its parasitoid *Plastygaster* sp. into producing thicker leaf galls that protect the parasitoid from hyperparasites [16]. Overall, examples of hypermanipulators are rare, but are important to identify and study as any ecological impacts associated with parasite manipulation of host phenotype may be modified in the presence of a hypermanipulator (e.g. [15]).

**Figure 1.**
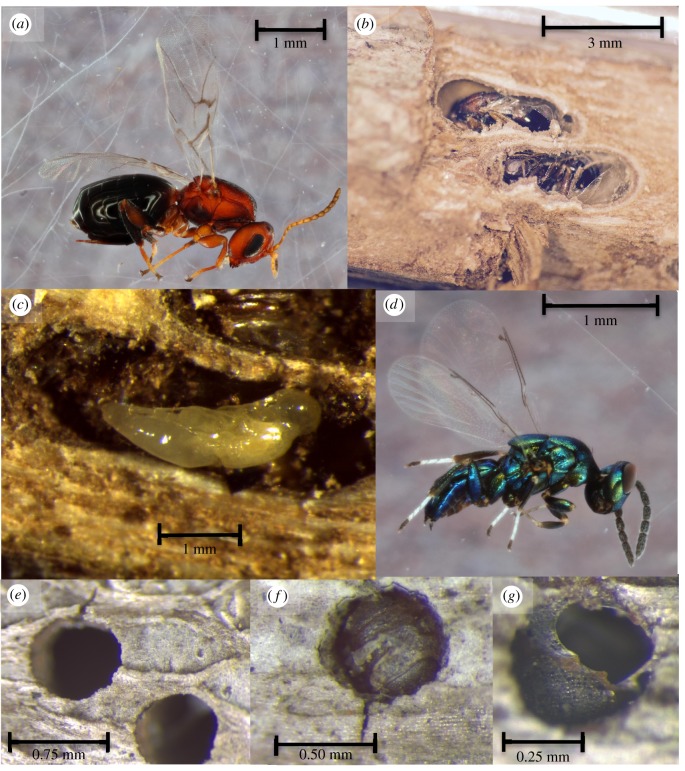
The crypt gall wasp *Bassettia pallida* infects sand live oaks, and induces the formation of ‘crypts’ in which the wasp will undergo development. *Bassettia pallida* infected by the crypt-keeper wasp *Euderus set* excavate small emergence holes that the host plugs with their head capsule prior to death. *Euderus set* emerges through the host's head capsule when it reaches its adult stage. (*a*) Adult *B. pallida*, (*b*) two dissected crypts containing adult *B. pallida*, (*c*) *E. set* pupa in a crypt made by *B. pallida*, (*d*) adult *E. set*, (*e*) emergence holes made by uninfected *B. pallida*, (*f*) emergence hole plugged by the head capsule of *B. pallida*, and (*g*) head-plugged hole with hole in *B. pallida*'s head capsule where *E. set* emerged.

Many apparent examples of manipulation lack direct experimental evidence of a fitness benefit to the parasite associated with the changed host phenotype [8]. Host phenotype may also change following infection owing to pathology or host compensation for infection [17], and in some systems where manipulation appeared to be occurring, it was later determined that the modified host phenotype did not in fact benefit the parasite (i.e. the host trait in question was probably not manipulated) [18]. Here, we both introduce a previously undocumented case of hypermanipulation (figure 1, and for an artist's illustration see the electronic supplementary material, figure S1) and manipulate the system to provide strong support that the observed changes in host behaviour benefit the parasite.

The host in this system is the asexual stage of the crypt gall wasp *Bassettia pallida*, a cynipid wasp found on sand live oaks (*Quercus geminata*) and southern live oaks (*Quercus virginiana*) in the southeastern USA [19–21]. Gall wasps induce changes in the morphology of their plant host, and in other gall wasp systems these morphological changes appear to benefit the host by protecting it from natural enemies [22,23]. The stem galls induced by *B. pallida* are known as crypts, which are tiny compartments in which *B. pallida* undergoes development before it excavates through the stem to emerge as an adult. While working in this system, we observed that many *B. pallida* had excavated emergence holes out of their crypts, but had died with their head plugging the holes. Dissections of 11 head-plugged crypts revealed clear evidence of a parasitoid in 10 cases, and one case where indirect evidence of a parasitoid was present. We never observed the egg stage of the parasitoid. However, in head-plugged crypts, we observed larval and pupal stages of the parasitoid residing partly within the crypt and partly within the host's body. This suggests that the manipulation of the host occurred sometime between the oviposition event and the larval parasitoid stage. The parasitoid appears to infect the adult host stage, as the parasitoid was never observed in a crypt with a larval or pupal host. The parasitoid was a previously undescribed eulophid parasitoid wasp, which we have named the ‘crypt-keeper wasp’ *Euderus set* (named after Set, the Ancient Egyptian God of evil and chaos) and is described elsewhere [24].

In this paper, we: (i) provide observational data on the timing of *E. set* emergence; (ii) quantify the fitness benefit to *E. set* of the ‘crypt-keeping’ phenomenon; and (iii) present museum and field data elucidating the temporal and geographical distribution of crypt-keeping manipulation.

## 2. Material and methods

### (a) Sample collection

Stems infected by *B. pallida* were collected from Inlet Beach, Florida (South Walton Beach County Park: 30.273517, −86.002499) in early August 2015 and mid-October 2015. Infected stems were found by visual inspection of sand live oaks (*Q. geminata*) within a 2 km radius. Stems were cut off the tree and stored in a plastic bag for transport to the laboratory.

### (b) Identification and natural history of host and parasite

The asexual generation of *B. pallida* Ashmead 1896 forms crypt-like stem galls hidden under the bark of host plants *Q. geminata* and *Q. virginiana* across the southeastern USA (FL, GA, AL, MS, LA and TX) [20,21]. Asexual generation adults emerge in the spring and adult emergence timing is synchronized with new leaf growth on its host plant, where the sexual generation is hypothesized to develop within galls on the midvein of new leaves (S. P. Egan 2016, unpublished data). The crypt-keeper wasp, *E. set*, is a parasitoid that appears to specialize on the asexual generation of the crypt gall wasp *B. pallida* on American live oaks in the genus *Quercus* and the subsection *Virentes* [19–21]. This is, to our knowledge, the first documented case of a member of the genus *Euderus* in this system [25].

### (c) Observational data on emergence timing

Stems collected from the field were brought back to Rice University, Houston, TX, USA, where they were processed under a dissecting microscope. All visible holes were numbered using a black marker, and hole status was noted as either ‘*B. pallida* emergence hole’ (an emergence hole that appeared to lead to an empty crypt), ‘head-plugged hole’ (an emergence hole plugged with a *B. pallida* head) and ‘*E. set* emergence hole’ (an emergence hole plugged with a *B. pallida* head, where the host's head capsule contained a hole from which a *E*. *set* had emerged) (figure 1). All hole states were re-examined following dissection at the end of the experiment (see Crypt dissections section).

Each stem was individually placed in a clear plastic cup, and a coffee filter secured by a rubber band covered the top of the cup. This housing allowed sunlight and air to penetrate the cup, but prevented the escape of insects that emerged from the stems. Cups were placed in bins and kept on a table in a shaded walkway exposed to natural conditions, but protected from rain and direct sunlight. The cups experienced natural daylight cycles and were exposed to naturally fluctuating temperature and humidity levels. Coffee filters were removed and the cups were misted with tap water once in February, and were misted weekly in March to mimic spring rain along the Gulf coast.

Cups were checked 5 days a week for emergences. Emerged insects were collected, placed in 96% ethanol and stored at −80°C for future analysis. When an emergence occurred, the stem was placed under a dissecting scope to look for new emergence holes or changes in the status of previous holes. The emergent insect was classified as *E. set*, *B. pallida*, or was identified as an inquiline or other parasitoid of *B. pallida*. These data provide us a first look at the timing of emergence for *B. pallida*, *E. set*, and the community of other parasitoids and inquilines associated with the asexual phase of this cynipid wasp.

We also observed differences in the diameters of emergence holes excavated by infected and uninfected *B. pallida,* which we discuss in the electronic supplementary material, Emergence hole analysis section.

### (d) Does the crypt-keeping phenomenon benefit *Euderus set*?

We hypothesized that the crypt-keeping phenomenon was an adaptive manipulation of *B. pallida* by *E. set*, and increased *E. set* fitness through one or two routes: (i) *E. set* benefits from *B. pallida* excavation of the emergence hole through the wood and bark of the tree as the parasitoid is less able or unable to excavate its own hole, and (ii) the *B. pallida* head-plug continues to seal the crypt from external abiotic conditions that may prohibit *E. set* development.

If *E. set* is able to manipulate *B. pallida* to create and subsequently plug an emergence hole, then *E. set* would only need to cut through or move aside the head of *B. pallida* to emerge. To test this hypothesis, we created a ‘reseal’ treatment where we resealed a subset of the head-plugged holes with bark, which would require *E. set* to excavate through both *B. pallida*'s head capsule and the bark to emerge as an adult. *Euderus set* emerging completely independent of host manipulation would need to chew through gall-associated plant material, typical woody tissue and bark to emerge. As chewing through the host's head capsule is probably less difficult than chewing through wood, this treatment may underestimate the difficulty *E. set* would experience if forced to emerge independently. Bark came from freshly harvested *Q. virginiana* stems on Rice University campus. A thin piece of bark was removed using a razorblade, and bark was soaked in tap water to make it pliable. Bark was placed over the head-plugged hole and secured using thin strips of standard laboratory labelling tape. The tape was placed so it held the bark over the hole without covering the hole. This hypothesis is specific to the adult stages of *E. set*, as only this stage would need to excavate an emergence hole. We predicted that survival would be highest for adult *E. set* in the ‘control’ treatment, and lower in the ‘reseal’ treatment, as *E. set* adults would need to emerge through *B. pallida*'s head capsule and bark in this treatment.

One hypothesized function of galls is to provide favourable abiotic conditions for the gall-former residing within [22,26]. To test this hypothesis, we identified a subset of head-plugged holes and created a ‘breach’ treatment by using an insect pin to poke a hole in the top of *B. pallida's* head. This breached the crypts and probably exposed the crypt contents to external conditions. However, this method could result in *E. set* mortality through two routes: by changing abiotic conditions, or because poking an insect pin into a crypt damaged *E. set*. To isolate the effect of mortality caused by poking an insect pin into a crypt with a developing parasitoid, we created a ‘breach & reseal’ treatment by covering a subset of the breach treatments with bark using the methods we described previously. We predicted that breaching the crypt would reduce the survival of all life stages of *E. set*, and *E. set* survival would be equal in control and breach & reseal treatments.

Manipulations were performed over two sessions. Head-plugged holes on stems collected during the August harvest session received treatments over five consecutive days in late August and early September, while stems harvested in October were treated over six consecutive days in early November. Survival status of *E. set* was determined by daily checks for *E. set* emergence as described earlier, and through dissections (see Crypt dissections section).

### (e) Temporal and geographical observational data on the crypt-keeping phenomenon

We encountered additional evidence of the crypt-keeping phenomenon in preserved specimens from the cynipid collections in the Smithsonian (US National Museum) and the American Museum of Natural History, as well as in the field. For museum specimens, we examined preserved *Bassettia* infested branches and quantified emergence holes that appeared to be a *Bassettia* emergence hole, a head-plugged hole or a *Euderus* emergence hole. Observations on museum specimens could not be confirmed with dissections. For field-collected specimens from other localities, holes were categorized, and hole status was confirmed by dissection (see Crypt dissections section).

### (f) Crypt dissections

We examined crypt contents during dissections by running a razorblade across the top of the stem until the inner crypt was revealed. As described previously, each crypt was categorized along a continuum as *B. pallida* emergence hole, head-plugged hole or *E. set* emergence hole. When *E. set* emerges or sometime after, the exoskeleton of *B. pallida* (including its head capsule) can fall back into the depths of the crypt, as many parasitoids will consume their host from the inside out leaving only the exoskeleton behind [27]. For this reason, any instance of an emergence hole containing *B. pallida* exoskeleton with no evidence of *E. set* remaining was deemed an *E. set* emergence hole. At this time, we also determined the fate of *E. set*. If remains of *E. set* were found in a crypt, then we recorded whether the remains were of the larval, pupal or adult stage. If we found an empty crypt containing *B. pallida* host remains, we determined that an adult *E. set* had emerged.

### (g) Statistical analyses

We performed all statistical analyses in RStudio v. 0.99.489 [28] running R v. 3.2.2 [29]. Models were created using the *glmer* function in the lme4 package [30]. Model comparisons were achieved using Akaike information criterion corrected (AICc) for small sample sizes [31], and model-averaged beta coefficients with 95% confidence intervals were obtained using the AICmodavg package [32].

We competed generalized linear mixed models (GLMMs) specifying a logit link function to examine how *E. set* survival was impacted by the treatments. All models contained a random intercept for stem ID. We competed four models to explore how our treatments impacted survival across all life stages: one null model (containing only the random intercept), one model containing a predictor for manipulation treatment (i.e. control, reseal, breach & reseal or breach), one model containing a predictor for stem harvest session, and one containing predictors for both manipulation treatment and stem harvest session. We did not include models with an interaction term, as sample sizes in August were low and we had no *a priori* reason to expect an interaction. We ran these models twice. The models were first run on a dataset restricted to only instances in which *E. set* had survived to adulthood, which allowed us to test our first hypothesis that *E. set* may have reduced survival when adults have to excavate their own emergence hole. We then ran the models looking at survival across all *E. set* life stages (i.e. including *E. set* that died as a larva, pupa or adult, as well as *E. set* that successfully emerged as adults), which allowed us to test our second hypothesis that breaching a crypt creates abiotic conditions unfavourable to *E. set* survival. Model-averaged predictor estimates and 95% confidence intervals were obtained using the *modavg* function.

## 3. Results

### (a) Observational data on emergence timing

From our Inlet Beach (FL) stems, 150 *E. set* emerged over the course of the project. While sporadic emergences occurred from mid-August through to late March, the main pulse of emergence (75% of emergences) occurred from mid-February through to mid-March. Some of these emergences occurred through crypts from which the head-plugging phenomenon was not observed, revealing that *E. set* is rarely able to emerge from crypts from which hosts have not excavated complete emergence holes. Two *B. pallida* emerged, with one emergence in mid-November and the other in mid-December. The rarity of *B. pallida* was not surprising, as adults should emerge in the spring synchronized with new leaf growth of their host plants [20]. Based on our observations, it appears that *E. set* undergoes development in the crypt during the time when the sexual stage of *B. pallida* resides in leaf galls, suggesting *E. set* only infects the asexual, crypt-making host stage.

A total of 39 other natural enemies (parasitoids or inquilines) emerged from the branches. Their emergences occurred throughout the duration of the experiment, and comprised 20% of the emergences we observed. These organisms were never associated with the crypt-keeping phenomenon, and all appeared to emerge from the crypts by creating their own emergence holes. The parasitoids included three species from the genus *Sycophila*, two species from genus *Ormyrus*, one each from the genera *Eurytoma*, *Acaenacis* and *Brasema*, and a platygastrid from the subfamily Platygastrinae. The inquilines included a species from the genus *Synergus* and another from the genus *Ceroptres*.

### (b) Does the crypt-keeping phenomenon benefit *Euderus set*?

**Figure 2.**
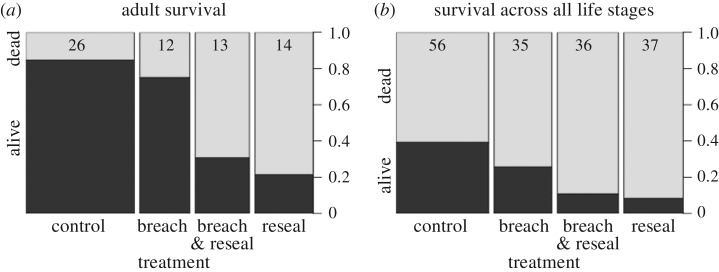
Proportion of *Euderus set* that survived or died in the control, breach, breach & reseal and reseal treatments of the manipulation experiment. (*a*) *Euderus set* survival restricted to instances where *E. set* survived to adulthood and (*b*) *E. set* survival across all life stages. Relative sample size is indicated by the width of each column, and absolute sample size is noted at the top of each column.

A total of 172 head-plugged holes were included in this experiment. The initial treatment sizes were 59, 37, 38 and 38 for the control, breach, reseal and breach & reseal treatments, respectively. Eight samples were removed from the analysis because the state of the crypt could not be determined unambiguously. The final sample size for analysis was 56, 35, 36 and 37 for control, breach, reseal and breach & reseal treatments, respectively. When the data were restricted to include only instances where *E. set* survived to adulthood, the sample sizes became 26, 12, 13 and 14 for the control, breach, breach & reseal and reseal treatments, respectively. Mortality was present across experimental treatments, including only 39% of all *E. set* surviving in the control group (figure 2), however, plant-mediated gall former death greater than 50% is common [33–35].

When we restricted our analysis to include only *E. set* that had survived to adulthood, we found that the model containing predictors for manipulation treatment and harvest date had the best fit and highest AICc weight (residual d.f. = 59; ΔAICc = 0, AICc weight = 0.8). The model containing only manipulation treatment received the rest of the AICc weight (residual d.f. = 60; ΔAICc = 2.83, AICc weight = 0.2). Breaching the crypts did not appear to reduce survival, while the breach & reseal and reseal treatments were associated with reliably negative log-likelihoods and thus reduced survival (table 1). The log-likelihood for the October harvest session was negative, but the 95% confidence interval included zero. Bringing stems into the laboratory later in the season either slightly reduced or did not impact the survival of adult *E. set*. While we did not look for interactions specifically, we note that running GLMMs to look for differences between the control and reseal treatments on the August and October datasets separately yielded qualitatively similar results.

**Table 1.** Model-averaged log-likelihoods and 95% confidence intervals (95% CI) for predictors of *E. set* adult survival, or survival across all life stages. (Treatments indicate whether head-plugged holes received no treatment (control), were breached to allow in ambient air (breach), were breached and then covered with a thin piece of bark (breach & reseal), or were covered with a thin piece of bark (reseal). Stems were collected during October and August harvest sessions.)

predictor	survived to adult stage		all life stages	
	estimate	95% CI	estimate	95% CI
intercept	2.67	0.82 to 4.52	0.78	−0.51 to 2.07
treatment: breach	−0.49	−2.23 to 1.26	−1.08	−2.29 to 0.13
treatment: breach & reseal	−2.78	−4.56 to -0.99	−2.28	−3.79 to −0.78
treatment: reseal	−3.4	−5.42 to −1.39	−2.95	−4.6 to −1.3
harvest session: October	−1.66	−3.31 to 0	−1.98	−3.33 to −0.62

The model that best predicted the survival of *E. set* across all life stages included terms for manipulation treatment and harvest session (residual d.f. = 158; ΔAICc = 0, AICc weight = 0.96). The model-averaged log-likelihoods for survival (table 1) were negative for breach & reseal and reseal treatments, and did not overlap with zero. The log-likelihood for the breach treatment was negative, but the 95% confidence interval overlapped with zero and so we are unable to say with certainty whether or not allowing ambient air into a crypt reduced *E. set* survival. The log-likelihood for the October session was negative, suggesting that *E. set* residing in stems collected at a later date were less likely to survive than those collected in August. While we did not look for interactions specifically, we note that running GLMMs to look for differences between the control, breach, and breach and reseal treatments on the August and October datasets separately yielded qualitatively similar results.

### (c) Temporal and geographical observational data on the crypt-keeping phenomenon

In addition to Inlet Beach (FL), we encountered evidence of the crypt-keeping phenomena on *Q. virginiana* and *Q. geminata* at four additional field sites across Florida, Mississippi and Texas (electronic supplementary material, table S1). In addition, we found historical samples in the cynipid collection at the Smithsonian, which suggests that this phenomenon has been ongoing since at least 1983 in this system involving live oaks, *B. pallida* and *E. set*.

In addition to observations in this specific system, we made observations of the crypt-keeping phenomenon outside of the live oak–*B. pallida–E. set* interaction. We observed branches with *Bassettia* emergence holes plugged with a *Bassettia* head capsule and *Bassettia* head capsules with *Euderus*-like emergence holes from field-collected samples on *Quercus nigra* in Houston, TX, USA, and on *Quercus lobata* and *Quercus douglasii* galled tissue from the cynpid collections at the American Museum of Natural History (electronic supplementary material, figure S2). This suggests that other members of the genus *Bassettia* may be manipulated by other species of *Euderus* across its range.

## 4. Discussion

The manipulation of host behaviour by parasites excites scientists across the spectrum, including neurobiologists, evolutionary biologists, physiologists and ecologists, as well as the general public [1,11,13,36–38], but few of these documented interactions provide a clear test of the fitness benefits for the parasite arising from parasite-associated host phenotypic changes [8]. In this study, we found that the crypt gall wasp *B. pallida* is infected by a previously undescribed parasitoid in the genus *Euderus.* Infection by *E. set* is associated with the host excavating a small emergence hole (figure 1 and see the electronic supplementary material, figure S1), and then plugging this hole with their head. This host behaviour benefits *E. set*, as *E. set* adults that had to excavate through bark themselves were about three times more likely to die trapped in the crypt relative to *E. set* that only needed to emerge through the host's head capsule (table 1 and figure 2). As parasitoids excavating through a breached host's head had similar survival to parasitoids that had to breach the host's head on their own (95% CIs for the breach & reseal treatment and the reseal treatment overlapped considerably), our experimental results suggest that the major cost of emergence is cutting through the bark. *Euderus set* emerging independently would need to cut through gall tissue of plant origin, typical woody tissue and bark, so our treatment (where *E. set* emerge through host cuticle and bark) may have underestimated the difficultly of emergence without host assistance. This system adds to a small, but growing set of examples of host–parasitoid systems where manipulation of host behaviour increases parasitoid fitness [9,14,39–42].

We provided clear evidence that *E. set* benefits from the crypt-keeping phenomenon. While this result strongly suggests that the manipulation is occurring, identifying the mechanism through which manipulation is induced would provide additional support. Energy drain is a common strategy used by parasites to manipulate their hosts [43], and *E. set* may have induced the crypt-keeping behaviour by draining enough energy that the host subsequently created a smaller excavation hole and died plugging the hole. Alternatively, injection of compounds into the host by the ovipositing *E. set* or secretion of compounds by the developing parasitoid may have induced the manipulation [13,44]. Teasing apart the specific induction methods beyond the presence of *E. set* is the next step, thus, we carefully considered alternative explanations to manipulation. One possible alternative is that this behavioural phenotype is circumstantial, in that the parasitoid is simply benefiting from a pre-existing host behaviour. During our dissections, we noticed instances where adult *E. set* had died trapped in a crypt in which *B. pallida* had not excavated an emergence hole, or had excavated a partial emergence hole that did not extend to the stem surface. This observation suggests there is variability in the ability of *E. set* to manipulate its host. However, the parasitoid could rather be searching out this behaviour and benefit from instances when its host engages in head-plugging behaviour. We think the ‘imperfect manipulation’ option is more likely for three critical reasons. First, traits of either host or parasite may influence the ability of the parasite to manipulate host phenotype [17,45,46], making variation in the expression of the manipulated phenotype likely (i.e. making it unsurprising that we observed 55 cases of what may be failed or incomplete manipulation). Second, the crypt-keeping phenomenon is tightly correlated with the presence of the parasitoid. In the 168 head-plugged holes in the experiment for which we were able to clearly examine crypt contents, in only four cases (i.e. 2% of cases) did we not find clear evidence of *E. set.* Additionally, dissections done in 2014 and 2015 found clear evidence of *E. set* in head-plugged holes in 37 of 39 instances and 10 of 11 instances, respectively. In cases where we did not find direct evidence of *E. set*, the parasitoid still may have been present, yet died at an early stage (e.g. when still an egg), decayed and left no trace. Given the high congruence between the crypt-keeping phenomenon and the presence of *E. set*, in order for the parasitoid to be benefiting from a pre-existing host behaviour rather than inducing the behaviour itself the ovipositing *E. set* would need to deposit her eggs in crypts where *B. pallida* was in the process of excavating an emergence hole, and the host would need to be paralysed or killed while excavating. When excavating *B. pallida* were unavailable, ovipositing *E. set* would instead oviposit in less desirable crypts in which *B. pallida* had not begun to excavate an emergence hole. We suspect this is not occurring because we would not expect to see a difference in emergence hole size between crypts containing *E. set* and those that do not (as described in the electronic supplementary material, Emergence hole analysis section) if *B. pallida* is simply being stopped in its tracks at some point in the excavation process. Additionally, we expect that *E. set* would be less tightly coupled with the crypt-keeping phenomenon if *E. set* were constrained to finding hosts in the process of emerging (rather than hosts simply residing in their crypts), but mechanistic work or observations during the *E. set* ovipositing period would be necessary to confirm. Finally, if taking advantage of pre-existing stuck gall wasps was a regular phenomenon, then we should see it in other gall-forming species in our live oak system—but we do not. In over 10 years of sampling, six different gall wasps species on live oaks (ranging from south Florida to Texas), we rarely see cynpids getting stuck (S. P. Egan 2006–2016 personal observation). When they do get stuck, it is usually between their head and thorax, and sometimes they get stuck completely intact and still in their gall. We never see the same head plug just below the surface of the bark with the head facing out, as we see when *E. set* infects *B. pallida*. Conservatively, our knowledge of this phenomenon in other gall-forming species arises from detailed observation of over 100 000 galls. In the rare instances we see non-*B. pallida* gall wasps get stuck in the gall or between their head and their larger thorax or abdomen, we have dissected these galls and never seen *Euderus* in its abdomen.

We found no evidence of reduced *E. set* survival in our breach treatment, despite our predictions that breaching the head-plugged crypts would result in reduced *E. set* survival owing to less than optimal abiotic conditions within breached crypts. This suggests that the head-plugging part of the crypt-keeping phenomena may not directly benefit *E. set*, and may simply arise because *B. pallida* manipulated into excavating small emergence holes get stuck. Alternatively, the benefit of head plugging may simply be that this behaviour prevents the host from emerging completely (which is critical as the parasitoid has not yet completed consuming the host), or the benefit may be something that we failed to measure. While keeping the cups outdoors allowed for semi-natural light and humidity conditions, maintaining the cups in this way may have excluded some ecologically relevant stressors. For example, in a previous year we observed a fairy wasp (Mymaridae, Chalcidoidea and Hymenoptera) emerge from infected stems, and fairy wasps have been reported to be hyperparasitoids of other Eulophid wasps (e.g. [47]). The hyperparasitoid may be an additionally important source of *E. set* mortality, but our experimental design did not allow for possible exposure to fairy wasps or other natural enemies of *E. set*. Manipulation to reduce hyperparasitism has been observed in other systems [16,39], and it is possible that head plugging makes it more difficult for hyperparasitoids to access the crypt (relative to breached or completely open crypts) and infect *E. set.* Alternatively, the presence of the head plug may actually provide to the fairy wasp an obvious visual cue to the presence of a developing *E. set.* Increased risk of encounters with natural enemies owing to manipulation has been observed in other systems [48–50], and increased hyperparasitoid infection rates due to the head-plugging phenomenon may constitute an indirect cost of manipulation.

The host plants for *B. pallida* (*Q. geminata* and *Q. virginiana*) are found in the southeastern USA, and it is likely that manipulation by *E. set* occurs throughout this range as our field excursions identified the crypt-keeping phenomenon occurring in Florida, Texas and Mississippi (electronic supplementary material, table S1). This phenomenon was also observed in museum samples from 1920 to 1948 on two different oak species found in California that harbour different *Bassettia* species (electronic supplementary material, figure S2). Parasitoids are often highly host specific [51], and *E. set* also appears to be host specific as it has not been observed infecting other gall wasp species found on *Q. geminata* or *Q. virginiana*, despite extensive work done on the gall formers of these oak trees [21,52]. We found no evidence of the crypt-keeping phenomenon in the literature or any similar manipulation of other gall wasps by their parasitoids, suggesting that the *Bassettia* species in California may be manipulated by a closely related species of *Euderus.* The undiscovered phenomenon in museum samples and field observations suggest it may be worth surveying the *Bassettia* throughout their range to explore the extent to which this gall wasp genus is manipulated by *Euderus* parasitoids. Further exploration of the extent to which *Euderus* manipulates its host may reveal instances in which this parasitoid could be useful as a form of biocontrol, as *Bassettia* infection can be associated with declines in their host tree (e.g. [53]), and other *Euderus* species infect insects known to be agricultural pests [25].

## 5. Conclusion

Few examples of hypermanipulation—where a phenotype-manipulating parasite is itself manipulated by a parasite—have been documented. Moreover, few studies confirm whether the host's changed behaviour increases parasite fitness, which is critical for a changed host behaviour to qualify as manipulation. Herein, we have described a novel case of hypermanipulation, in which the crypt gall wasp *B. pallida*, a phenotypic manipulator of its host plant, is manipulated by the Eulophid parasitoid, the crypt-keeper wasp *E. set*, and clearly demonstrate that the host's changed behaviour increases parasitoid fitness. Moreover, using museum specimens, literature review and observational data from the field, we have highlighted a possible undocumented complex interaction that may be continental in scale (close to 600 Eulophid species in North America; [54]). This previously undocumented phenomenon may prove economically important, as many Eulophid parasitoids attack and(or) serve as biocontrol agents for major agricultural pests, including apple leafminers, Colorado potato beetle, asparagus beetle, fruit tree leafrollers and western flower thrips [54].

## Supplementary Material

Emergence Hole Analysis

## Supplementary Material

Figures and Tables

## Data accessibility

Our analysis of emergence hole diameter and two figures and a table are available in the electronic supplementary material. Data and R code for analysing the data are available in Dryad: http://dx.doi.org/10.5061/dryad.dm383 [55].
