# Supplementary material for: Tales from the crypt: a parasitoid manipulates the behaviour of its parasite host
Source: Proc Biol Sci. 2017 Jan 25;284(1847):20162365. doi: 10.1098/rspb.2016.2365 (PMC5310038; doi:10.1098/rspb.2016.2365)

Tales From The Crypt:

A Parasitoid Manipulates the Behaviour of Its Parasite Host

DOI: 10.1098/rspb.2016.2365

Kelly L. Weinersmith,[1*] Sean M. Liu,[1] Andrew A. Forbes,[2] and Scott P. Egan[1]

[1]Department of BioSciences, Rice University, MS-140, 6100 Main Street, Houston, TX 77005; [2]Department of Biology, University of Iowa, Iowa City, IA 52242

*Author for Correspondence: Kelly Weinersmith (e-mail: Kelly.Weinersmith@gmail.com)

Supplementary Material

Analysis of Emergence Hole Diameters

2. Methods

(a) Emergence hole diameter analysis

Hole diameter appeared to be associated with the identity of the emergent animal. Two measurements of hole diameter were made perpendicular to each other under a Leica M125 Stereo microscope, and the two measurements were averaged. We attempted to measure all holes on the stems collected during the August harvesting session, and a subset of holes on stems from the October harvesting session (focusing on stems that contained all three hole types). Holes were skipped if stem shape prohibited us from getting the hole in an appropriate plane to permit accurate measurements of hole diameter under the microscope.

We performed all statistical analyses in RStudio Version 0.99.489 [1] running R Version 3.2.2 [2]. Models were created using the *lmer* function in the lme4 package [3]. Model comparison using AICc, and model averaged beta coefficients with 95% confidence intervals were obtained using the AICmodavg package [4].

To explore how the infection status of *B. pallida* influenced the diameter of the associated emergence hole, we fit 4 linear mixed effects models. An identification code specific to the stem on which a hole was measured was included as a random intercept in all models, as *B. pallida* on the same stem may lack independence. The competing models included a null model (random intercept only), and the other 3 models contained either a term for *B. pallida* infection status, a term indicating whether the stems were harvested from Inlet Beach during the first or second sampling session, or both terms. Model-averaged predictor estimates and 95% confidence intervals were obtained using the *modavg* function.

3. Results

(a) Emergence hole diameter analysis

We measured the diameter of a total of 889 holes (404 holes associated with infected *B. pallida*, and 485 associated with uninfected *B. pallida*) across 57 stems. 465 holes were measured on stems collected during the August harvest session, and 424 were measured on stems collected during the October harvest session. The highest ranking model had an AICc weight of 0.73, and contained only a term for host infection status (Table 1, see below). The second highest ranking model had an AICc weight of 0.27, and contained terms for host infection status and harvest session. Parameter estimates, averaged across all models, were 0.5 (95% CI: 0.48 to 0.52) for the intercept, 0.18 for uninfected hosts (95% CI: 0.17 to 0.20), and 0.00 (95% CI: -0.03 to 0.03) for the October sampling session. Thus, infected hosts create emergence holes that are on average 0.18 mm smaller in diameter than those associated with crypts harboring uninfected *B. pallida* (Figure 1, see below). *B. pallida* head capsule widths were ~0.3 to 0.5mm, so infected *B. pallida* excavate emergence holes that are about 50% smaller relative to the width of their head. Harvest session likely did not influence emergence hole diameters (Figure 1).

4. Discussion

Gall wasps are frequently infected by a diverse community of parasitoids and inquilines [5–7]. In our system, we observed 12 natural enemies associated with *B. pallida*. While *E. set* accounted for the majority of the parasitoids emerging from the stems we collected, 20% of the emergences were comprised of other community members. While *E. set* appears to consume the adult stage of *B. pallida* and leaves evidence of its presence in the form of *B. pallida* exoskeleton scattered around the crypt, most parasitoids consume the immature host stages [8–10], and would be less likely to leave behind evidence of their presence if the parasitoid survived to emergence. This suggests that holes excavated by parasitoids other than *E. set* were likely mischaracterized as holes associated with uninfected *B. pallida*. As parasitoids are typically equal in size or smaller than their hosts [11], these parasitoids likely excavated smaller emergence holes, driving down our estimate of hole diameter for crypts associated with uninfected *B. pallida*. These mischaracterized holes may explain the small average diameter outliers in our dataset associated with uninfected *B. pallida*. Thus, our conclusion that infected *B. pallida* excavate smaller emergence holes than their uninfected conspecifics is likely sound, and the effect is greater than what we report here.

References

1. RStudio Team 2015 *RStudio: Integrated Development for R.* Boston, MA: RStudio, Inc.

2. R Development Core Team 2015 *R: A language and environment for statistical computing*. Vienna, Austria: R Foundation for Statistical Computing.

3. Bates, D., Maechler, M., Bolker, B. & Walker, S. 2015 Fitting linear mixed-effects models using lme4. *J. Stat. Softw.* **67**, 1–48. (doi:10.18637/jss.v067.i01)

4. Mazerolle, M. J. 2016 *AICcmodavg: Model selection and multimodel inference based on (Q)AIC(c)*.

5. Stone, G. N. & Schönrogge, K. 2003 The adaptive significance of insect gall morphology. *Trends Ecol. Evol.* **18**, 512–522.

6. Stone, G. N., Schönrogge, K., Atkinson, R. J., Bellido, D. & Pujade-Villar, J. 2002 The population biology of oak gall wasps (Hymenoptera: Cynipidae). *Annu. Rev. Entomol.* **47**, 633–668. (doi:10.1146/annurev.ento.47.091201.145247)

7. Forbes, A. A., Hall, M. C., Lund, J., Hood, G. R., Izen, R., Egan, S. P. & Ott, J. R. 2015 Parasitoids, hyperparasitoids, and inquilines associated with the sexual and asexual generations of the gall former, *Belonocnema treatae* (Hymenoptera: Cynipidae). *Ann. Entomol. Soc. Am.* , sav112. (doi:10.1093/aesa/sav112)

8. Redfern, M. & Askew, R. R. 1992 *Plant Galls*. Slough, England: The Richmond Publishing Co. Ltd.

9. Godfray, H. C. J. 1993 *Parasitoids*. Princeton, N.J: Princeton University Press.

10. Tagawa, J. & Fukushima, H. 1993 Effects of host age and cocoon position on attack rate by the hyperparasitoid, *Eurytoma* sp. [Hym.: Eurytomidae], on cocoons of the parasitoid, *Cotesia (=Apanteles) glomerata* [Hym.: Braconidae]. *Entomophaga* **38**, 69–77. (doi:10.1007/BF02373141)

11. Lafferty, K. D. & Kuris, A. M. 2002 Trophic strategies, animal diversity and body size. *Trends Ecol. Evol.* **17**, 507–513.

Table 1: Ranking of models predicting the average diameter of emergence holes. All models contain a random intercept for stem ID. Host Infection Status refers to whether the *Bassettia pallida* host associated with an emergence hole was or was not infected by *Euderus set*. Harvest Session refers to whether the stems for the experiment were collected during the August or October stem harvest sessions.

Model	Residual df	ΔAICc	Weight
Average Hole Diameter = (1|StemID)			
+ Host Infection Status	885	0	0.73
+ Host Infection Status + Harvest Session	884	1.99	0.27
null	886	435.36	0.00
+ Harvest Session	885	436.98	0.00

Figure 1: Boxplots showing the average diameter of emergence holes (mm), (A) associated with *B. pallida* hosts infected by *E. set* and uninfected *B. pallida* and (B) from the August and October stem harvest sessions. The center line in the boxplot indicates median values, and the top and bottom of the boxes indicated first and third quartiles. Whiskers indicate maximum and minimum data points, with open circles indicating outliers that fall greater than 1.5 times beyond the interquartile range.

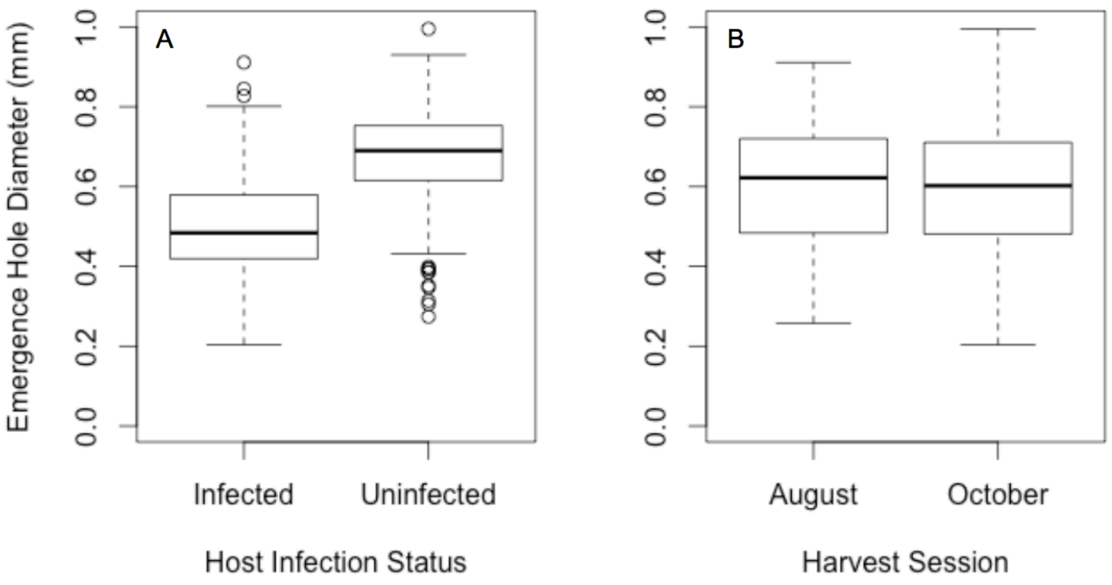

Supplement: Emergence Hole Analysis [file rspb20162365supp1.pdf]
