# Supplementary material for: Tales from the crypt: a parasitoid manipulates the behaviour of its parasite host
Source: Proc Biol Sci. 2017 Jan 25;284(1847):20162365. doi: 10.1098/rspb.2016.2365 (PMC5310038; doi:10.1098/rspb.2016.2365)
Supplement: Figures and Tables [file rspb20162365supp2.pdf]

Tales From The Crypt:

A Parasitoid Manipulates the Behaviour of Its Parasite Host

DOI: 10.1098/rspb.2016.2365

Kelly L. Weinersmith,[1*] Sean M. Liu,[1] Andrew A. Forbes,[2] and Scott P. Egan[1]

[1]Department of BioSciences, Rice University, MS-140, 6100 Main Street, Houston, TX 77005; [2]Department of Biology, University of Iowa, Iowa City, IA 52242

*Author for Correspondence: Kelly Weinersmith (e-mail: Kelly.Weinersmith@gmail.com)

Supplemental Figure 1. The crypt-keeping phenomenon. *(a)* The crypt gall wasp, *Bassettia pallida*, oviposits an egg into new stems on *Quercus virginiana* and *Quercus geminata*. *(b)* A compartment known as a "crypt" is induced in the tree stem, and *B. pallida* will hatch and undergo development in this crypt. *(c)* If *B. pallida* remains uninfected and survives to becomes an adult, it excavates an emergence hole through the stem and leaves the crypt. *(d)* The crypt-keeper wasp, *Euderus set*, oviposits its egg into a crypt made by *B. pallida*. *(e)* The *B. pallida* host excavates a small emergence hole, plugs the hole with its head capsule, dies, and is consumed by the developing *E. set*. *(f)* Overhead view of *B. pallida*'s head plugging an emergence hole. *(g)* When *E. set* reaches adulthood is emerges through its host's head capsule and leaves the crypt. *(h)* Overhead view of *B. pallida's* head capsule breached by an emerging *E. set* adult. All artwork by Boulet (http://english.bouletcorp.com/).

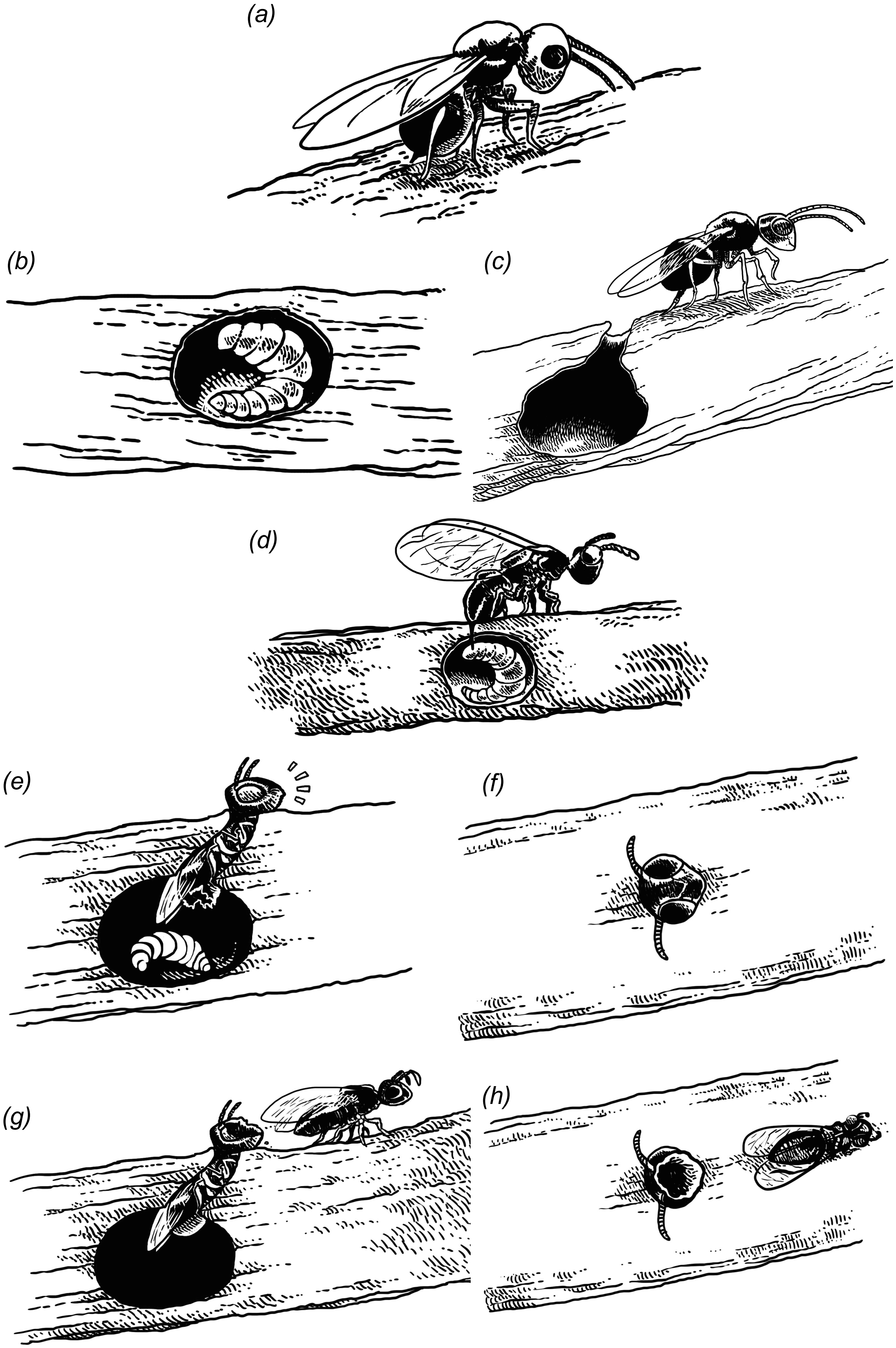
(a)
(b)
(c)
(d)
(e)
(f)
(g)
(h)

Supplemental Figure 2: The head-plugging phenomenon found on samples from the American Museum of Natural History. (A) Head capsule of *Bassettia ligni* plugging an emergence hole on a stem from *Quercus lobota.* (B) *B. ligni* head capsule with evidence of *Euderus* emergence on stem from *Q. lobota*. (C) Head capsule of *B. ligni* plugging an emergence hole on a stem from *Q. douglasii*.

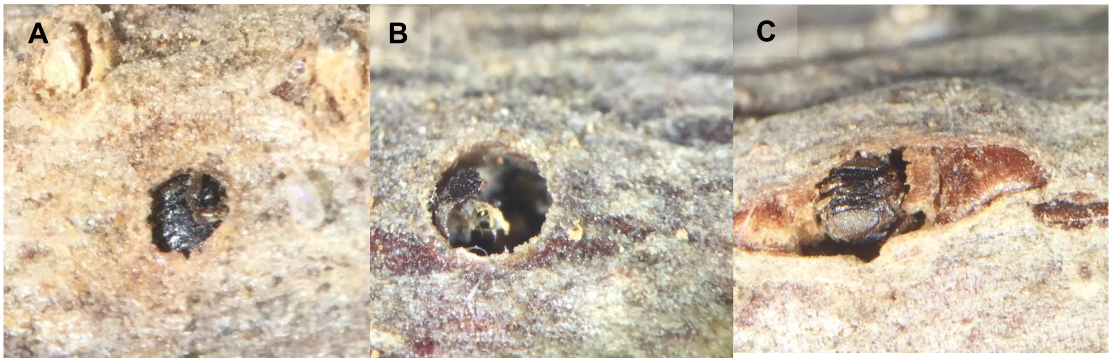

Supplemental Table 1: Additional observations of the crypt-keeping phenomenon. Year the sample was collected, locality, host plant, host species, and the number of times we observed holes plugged by a *Bassettia* head capsule (head-plugged), holes from which *Euderus* presumably emerged through the head of *Bassettia* (*Euderus* emergence), and holes from which *Bassettia* emerged successfully (*Bassettia* emergence) are indicated. Hole status was confirmed with dissection in the field collected samples, but not in samples collected from the Smithsonian Institute (SI) or the American Museum of Natural History (AMNH).

Year	Locality	Host plant	Host species	Head-plugged	*Euderus* emergence	*Bassettia* emergence
Live oak – *Bassettia pallida* – *Euderus bassettia* system						
2015	Gautier, MS	*Q. virginiana*	*B. pallida*	7	2	10
2015 & 2016	Humble, TX	*Q. virginiana*	*B. pallida*	7	37	552
2016	Rice University, TX	*Q. virginiana*	*B. pallida*	4	0	16
2015	Lake Lizzie, FL	*Q. geminata*	*B. pallida*	7	6	91
2015	Ochlocknee River, FL	*Q. geminata*	*B. pallida*	7	1	22
1983 (SI)	Broward Co., Ft. Lauderdale, FL	*Q. virginiana*	*B. pallida*	7	7	158
Other oak – *Bassettia* systems						
2015 & 2016	Houston, TX	*Q. nigra*	Unknown	2	2	49
1948 (AMNH)	Stanford, CA	*Q. lobata*	*B. ligni*	4	3	66
1920 (AMNH)	Galt, CA	*Q. douglasii*	*B. ligni*	2	2	84
